# Supplementary figures and images for: Multiple independent losses of crossover interference during yeast evolutionary history
Source: PLoS Genet. 2024 Sep 26;20(9):e1011426. doi: 10.1371/journal.pgen.1011426 (PMC11460703; doi:10.1371/journal.pgen.1011426)

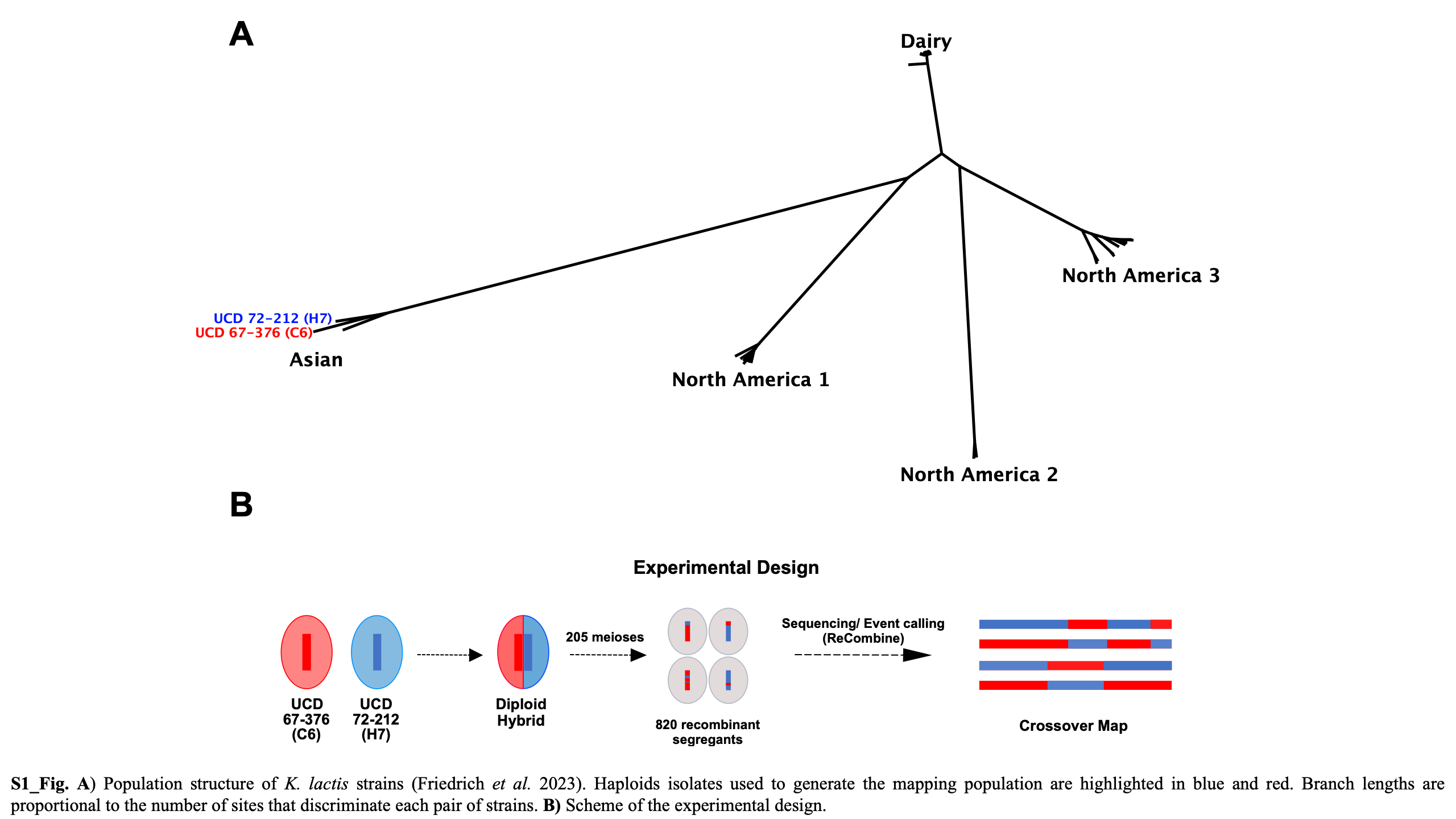

Supplement: S1 Fig — A) Population structure of K. lactis strains [48]. Haploids isolates used to generate the mapping population are highlighted in blue and red. Branch lengths are proportional to the number of sites that discriminate each pair of strains. B) Scheme of the experimental design. (TIFF) [file pgen.1011426.s008.tiff]

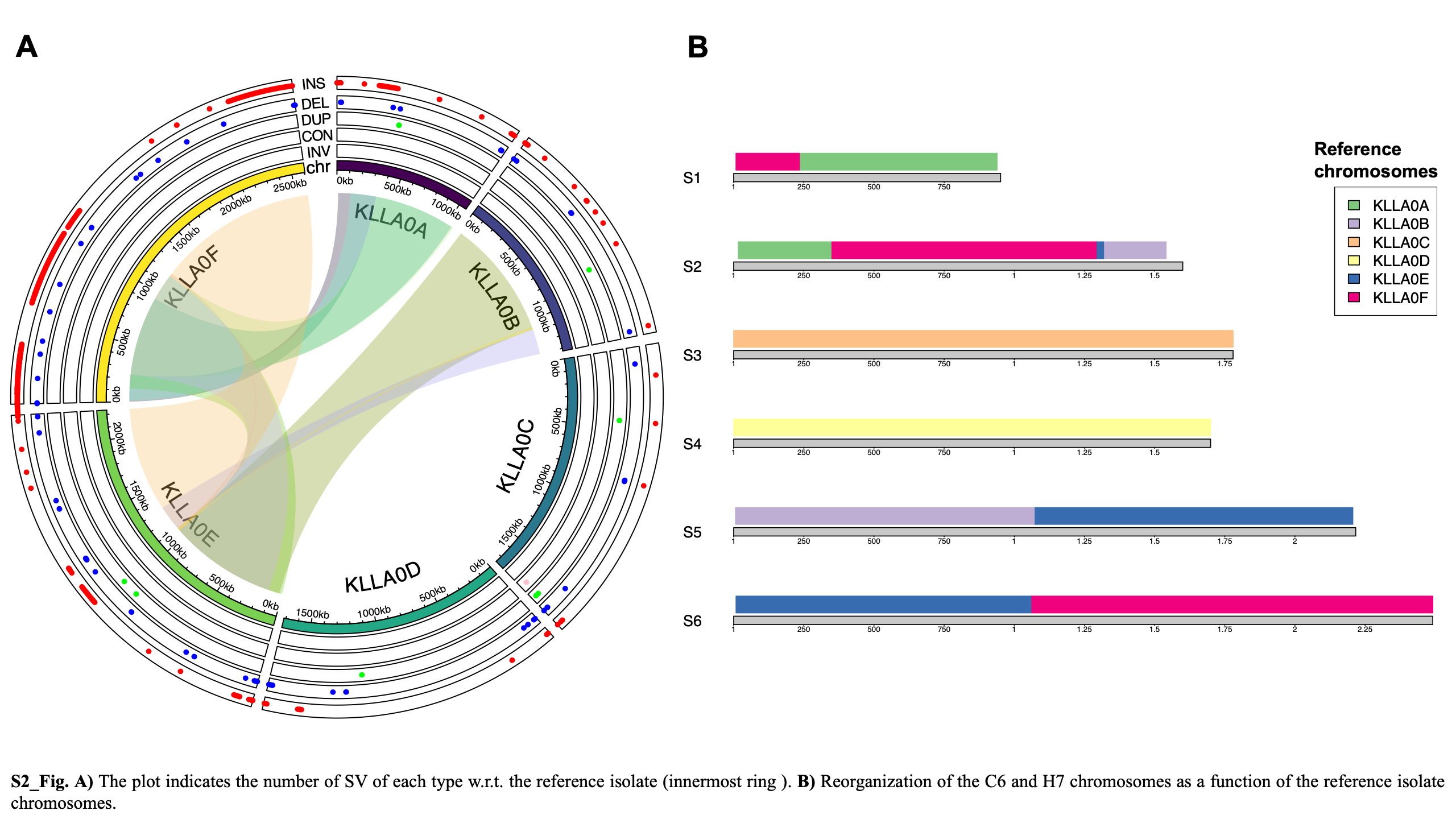

Supplement: S2 Fig — A) The plot indicates the number of SV of each type w.r.t. the reference isolate (innermost ring). B) Reorganization of the C6 and H7 chromosomes as a function of the reference isolate chromosomes. (TIFF) [file pgen.1011426.s009.tiff]

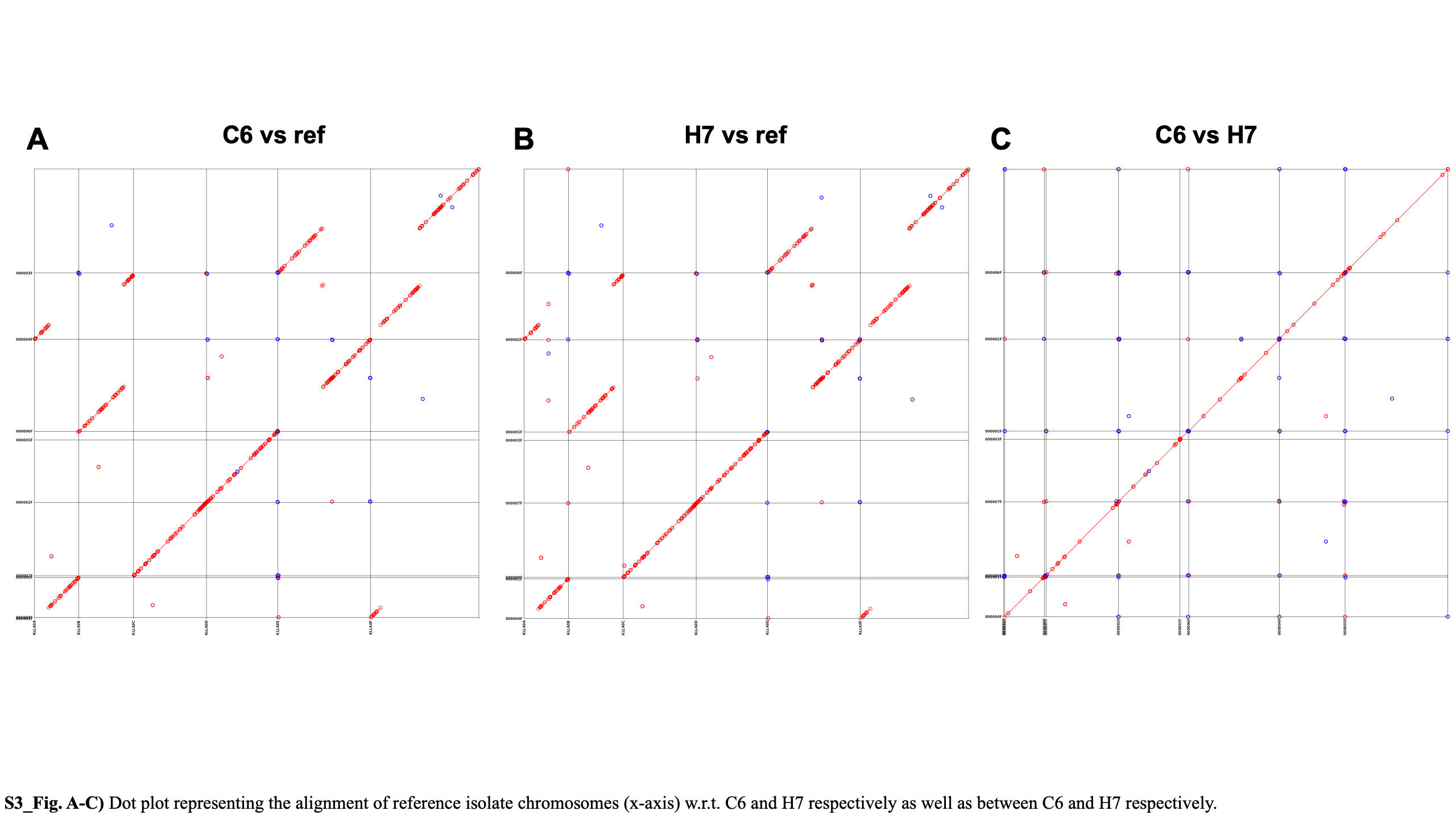

Supplement: S3 Fig — A-C) Dot plot representing the alignment of reference isolate chromosomes (x-axis) w.r.t. C6 and H7 respectively as well as between C6 and H7 respectively. (TIFF) [file pgen.1011426.s010.tiff]

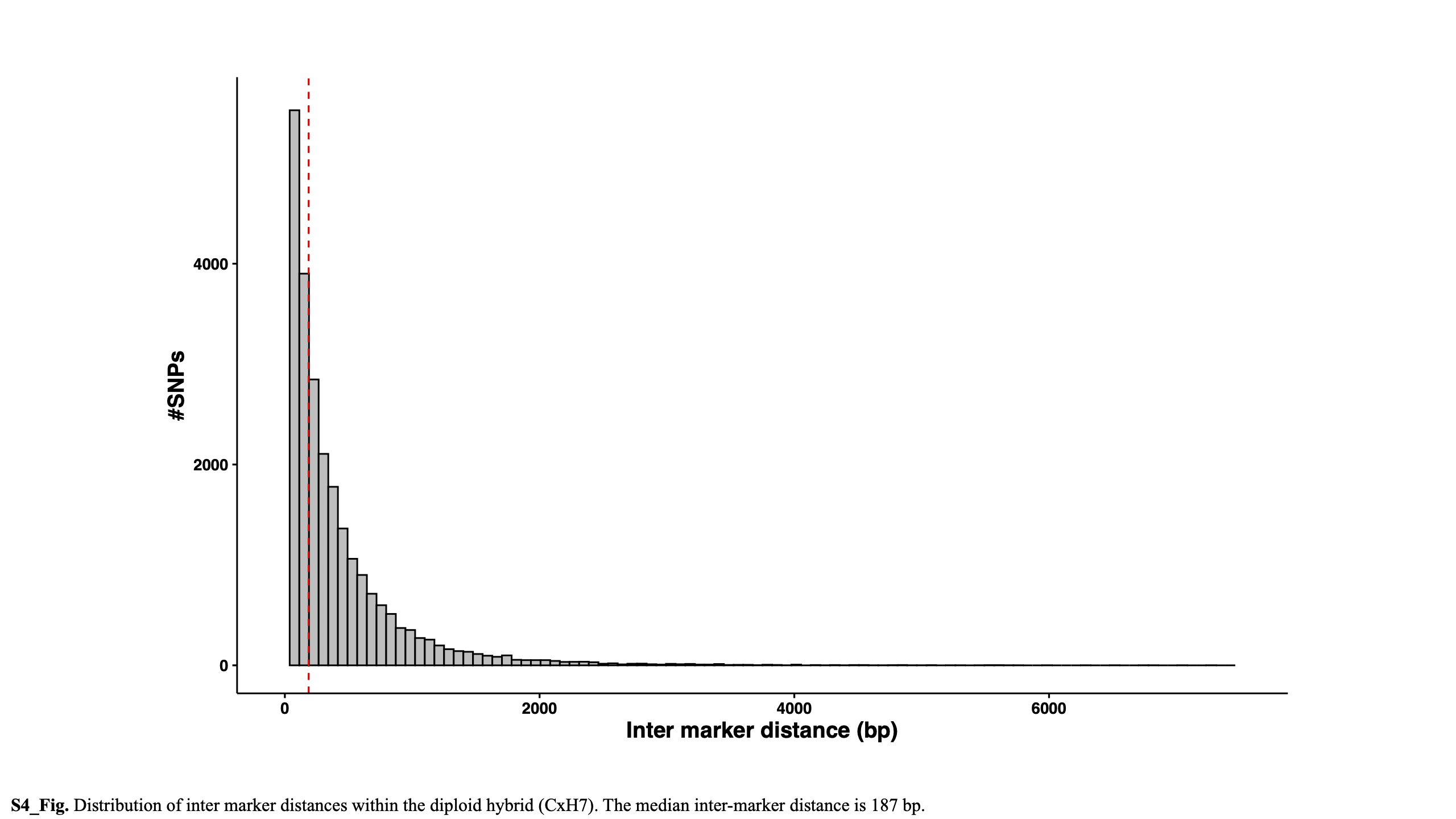

Supplement: S4 Fig — Distribution of inter marker distances within the diploid hybrid (CxH7). The median inter-marker distance is 187 bp. (TIFF) [file pgen.1011426.s011.tiff]

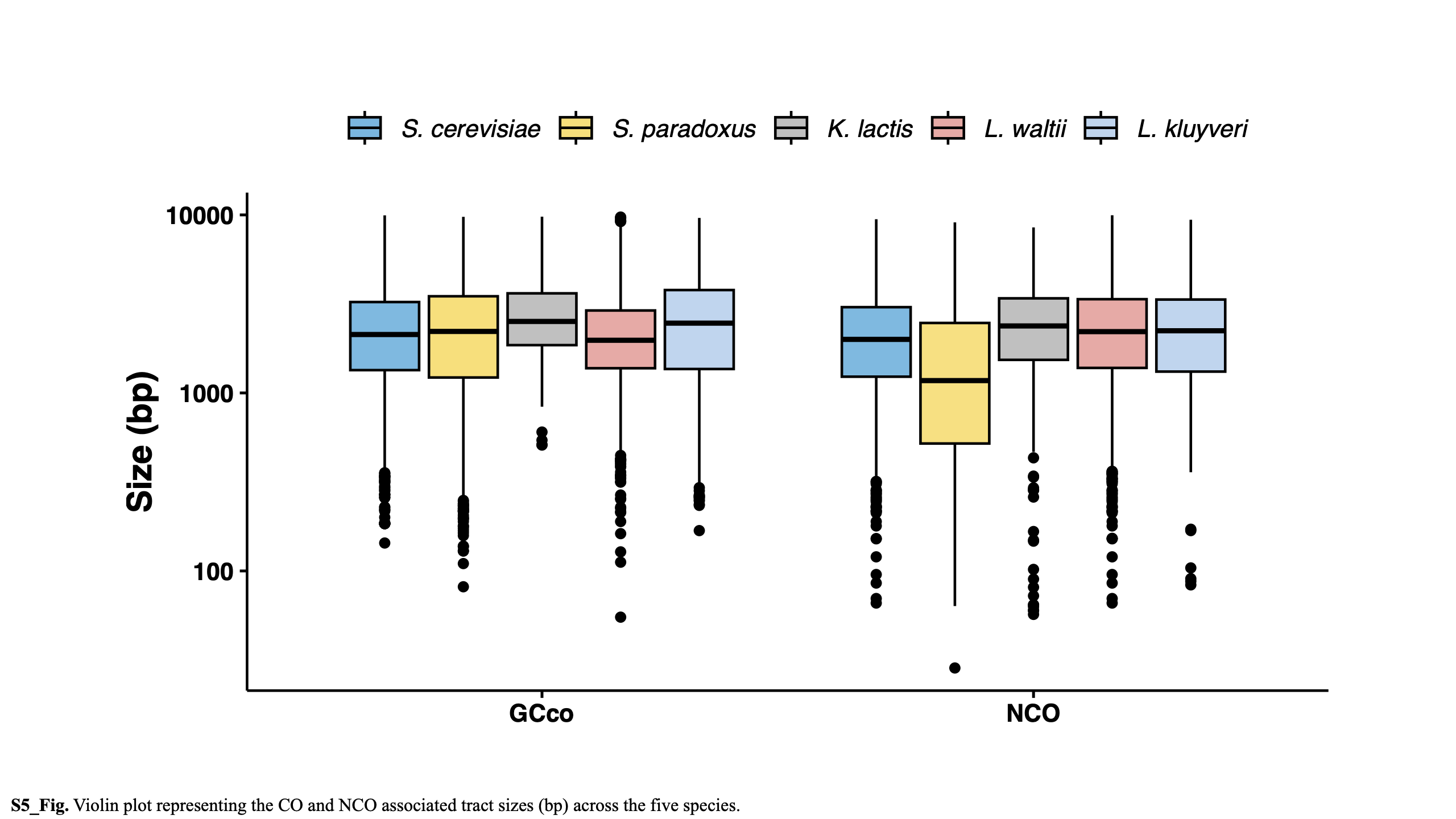

Supplement: S5 Fig — Violin plot representing the CO and NCO associated tract sizes across the five species. (TIFF) [file pgen.1011426.s012.tiff]

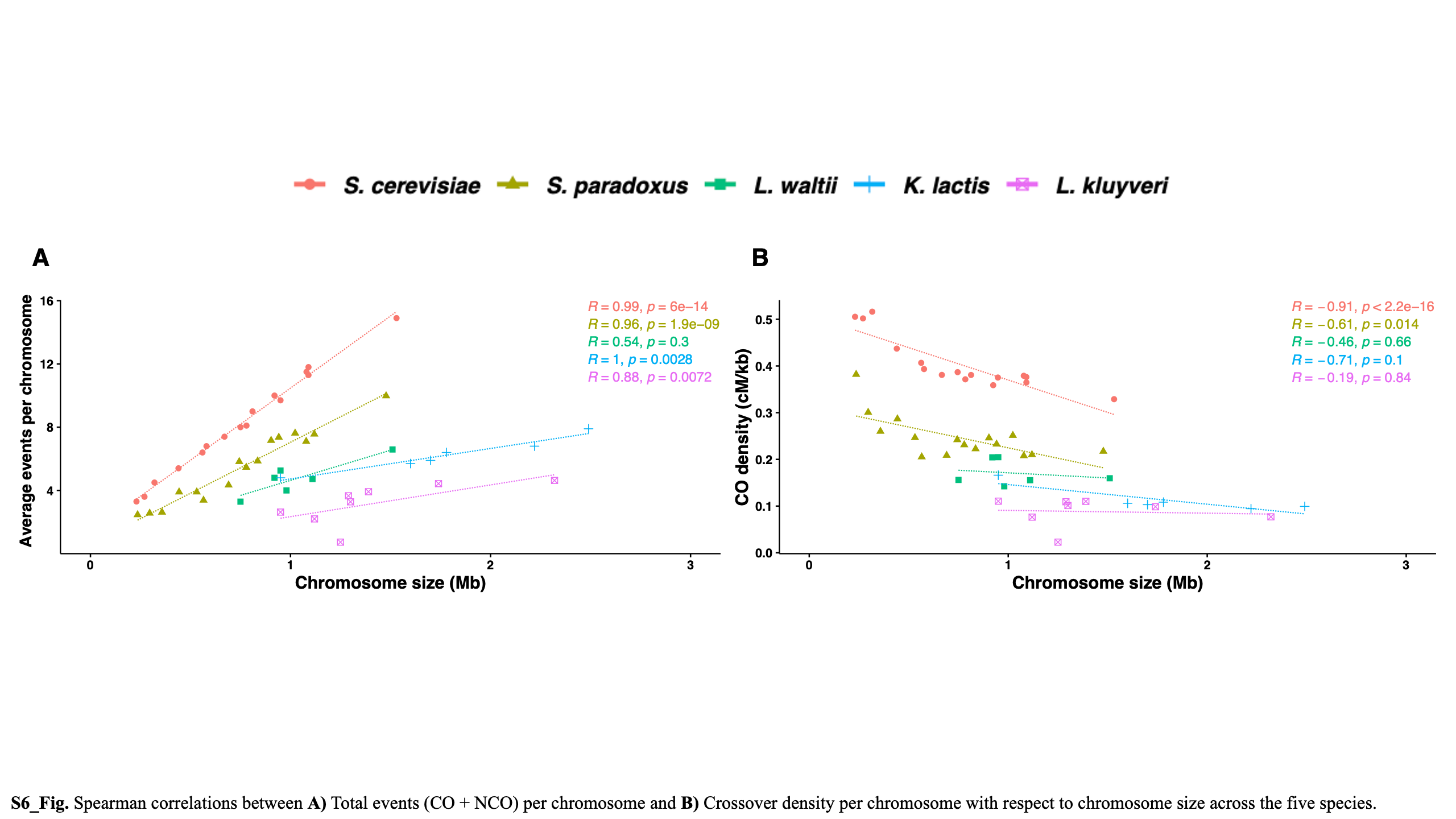

Supplement: S6 Fig — Spearman correlations between A) Total events (CO + NCO) per chromosome and B) Crossover density per chromosome with respect to chromosome size across the five species. (TIFF) [file pgen.1011426.s013.tiff]

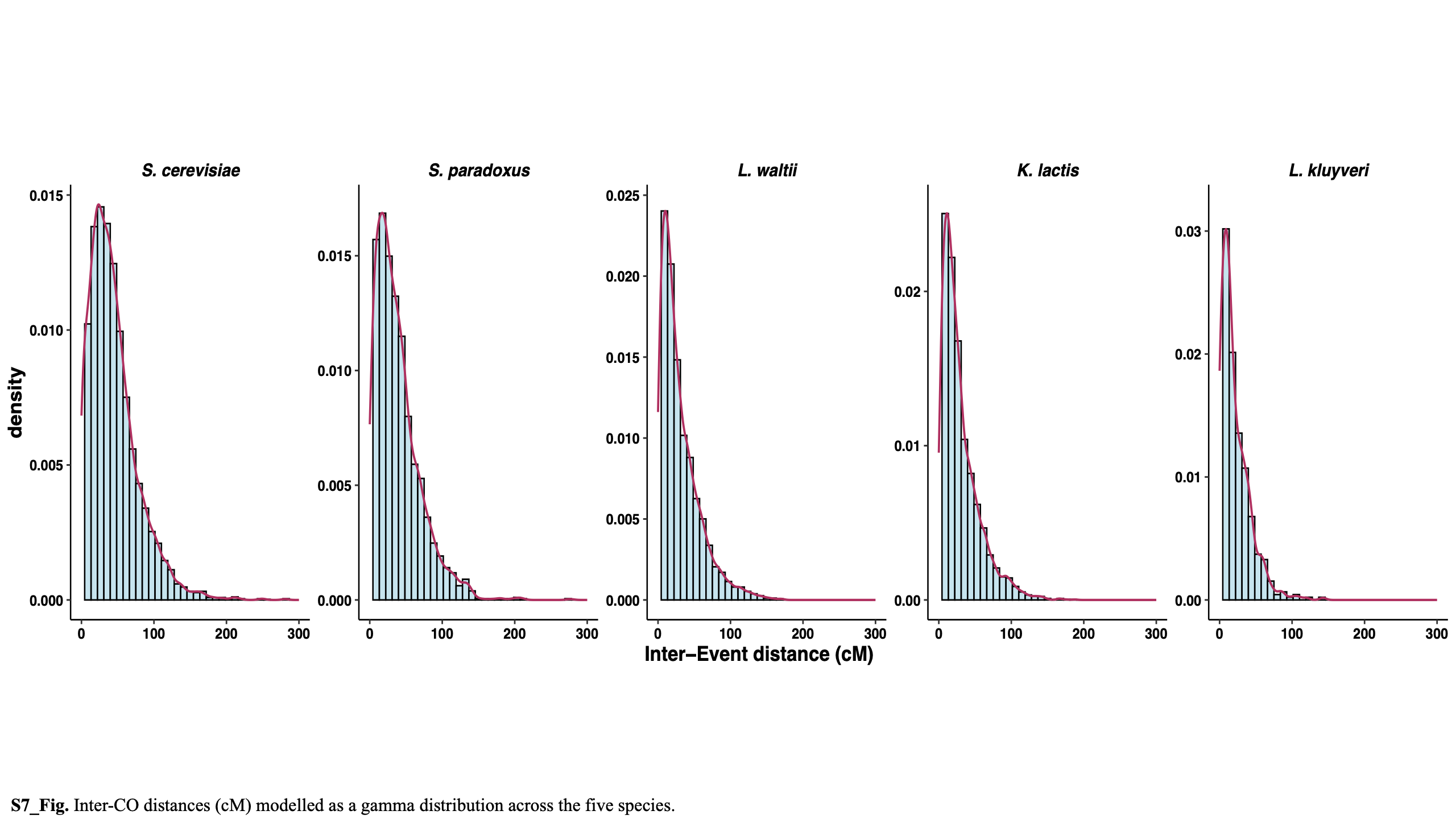

Supplement: S7 Fig — Inter-CO distances (cM) modelled as a gamma distribution across the five species. (TIFF) [file pgen.1011426.s014.tiff]

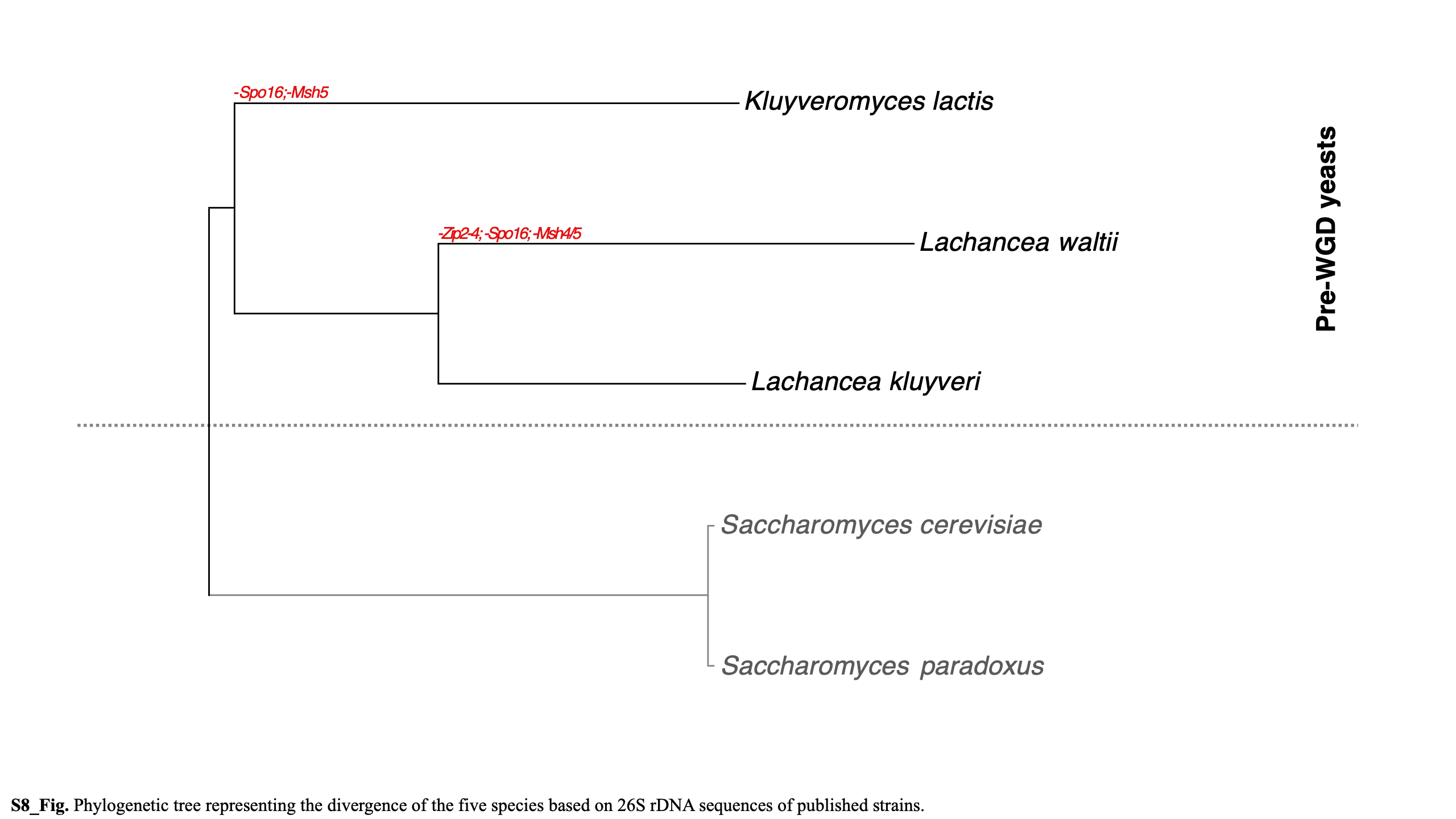

Supplement: S8 Fig — Phylogenetic tree representing the divergence of the five species based on 26S rDNA sequences of published strains. (TIFF) [file pgen.1011426.s015.tiff]

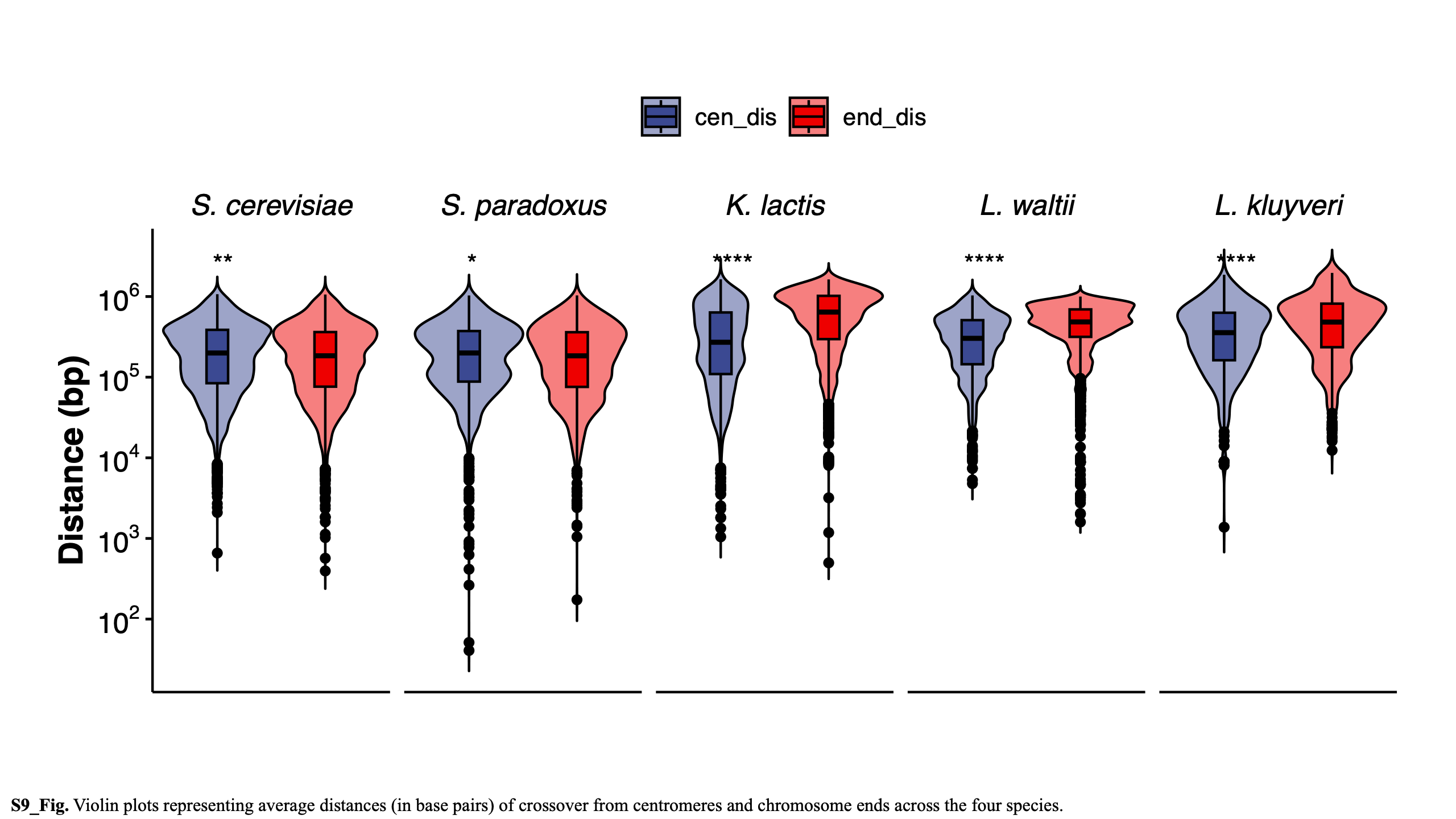

Supplement: S9 Fig — Violin plots representing average distances (in base pairs) of crossover from centromeres and chromosome ends across the four species. (TIFF) [file pgen.1011426.s016.tiff]

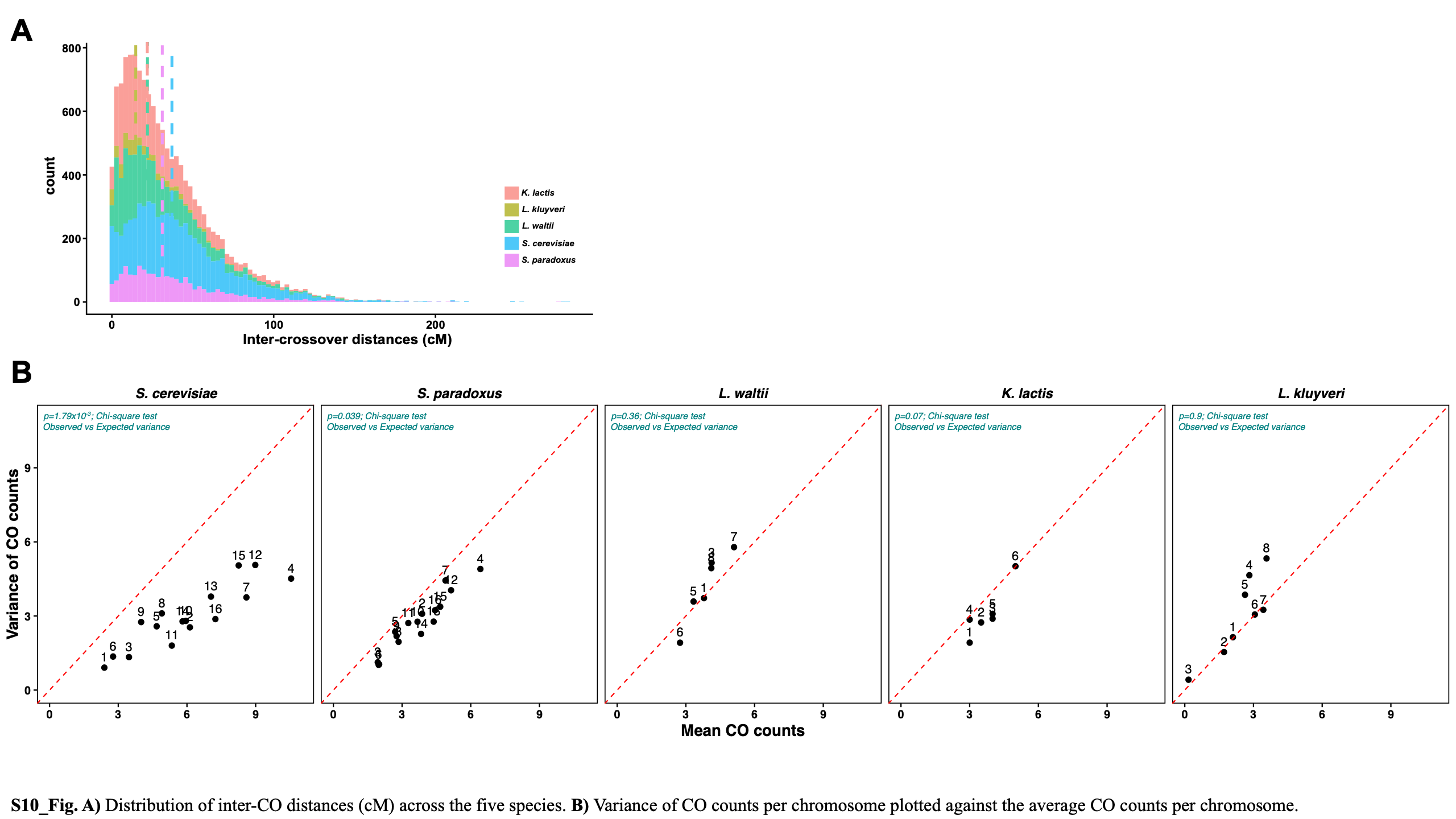

Supplement: S10 Fig — A) Distribution of inter-CO distances (cM) across the five species. B) Variance of CO counts per chromosome plotted against the average CO counts per chromosome. (TIFF) [file pgen.1011426.s017.tiff]

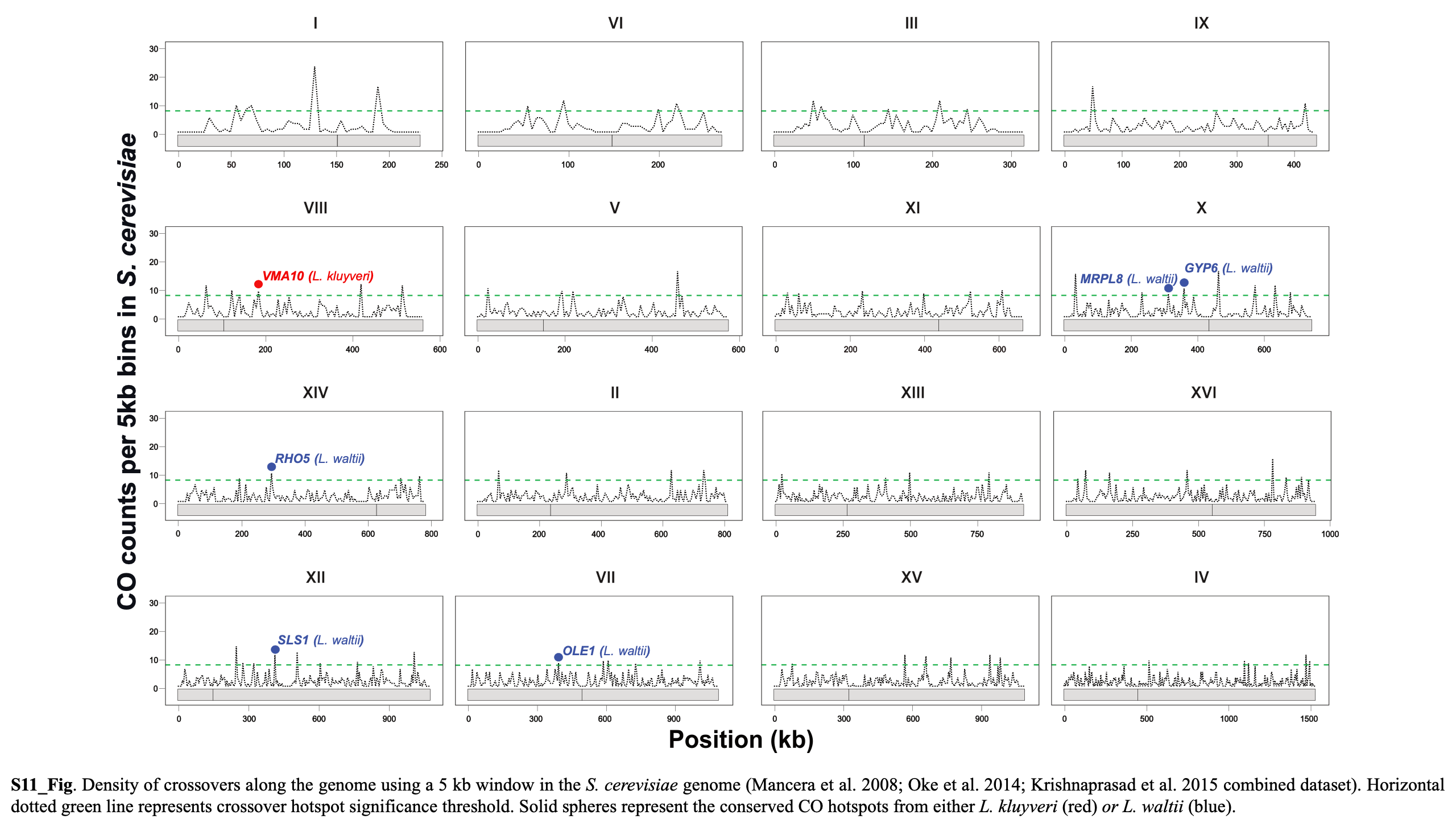

Supplement: S11 Fig — Density of crossovers along the genome using a 5 kb window in the S. cerevisiae genome (Mancera et al. 2008; Oke et al. 2014; Krishnaprasad et al. 2015 combined dataset) [12,50,57]. Horizontal dotted green line represents crossover hotspot significance threshold. Solid spheres represent the conserved CO hotspots from either L. kluyveri (red) or L. waltii (blue). (TIFF) [file pgen.1011426.s018.tiff]
