# Supplementary material for: Multiple independent losses of crossover interference during yeast evolutionary history
Source: PLoS Genet. 2024 Sep 26;20(9):e1011426. doi: 10.1371/journal.pgen.1011426 (PMC11460703; doi:10.1371/journal.pgen.1011426)
Supplement: Attachment — Submitted filename: 145792_1_rebuttal_3073125_sg3xs1.docx [file pgen.1011426.s019.docx]

**Author responses**

**Reviewer #1 (Evidence, reproducibility and clarity (Required)):**

In their manuscript, Dutta and colleagues compared the meiotic recombination landscapes between five budding yeast species. In the first part of the work, the authors constructed a high-resolution map of meiotic recombination events in Kluyveromyces lactis supported by high-quality genome assemblies for two strains of this yeast. Then, partially repeating their CO and NCO mapping strategy, they compared a number of meiotic recombination parameters between the five species (sometimes three, depending on the quality of the data for each species). They particularly focused on key parameters for meiotic recombination, such as crossover interference and homeostasis and obligate crossover. Although the analysis is interesting, it is underdeveloped in many places and lacks the general conclusions regarding the evolution of recombination and the broader perspective that would be expected from a comparison of these phenomena in budding yeasts.

[R] Tackling the evolution of recombination is ambitious. Here, with a dataset of five species, it is hard to draw strong evolutionary conclusions besides the variations in the crossover (CO) landscapes and the control of CO formation that we observed, which is already significant. The multiple losses of CO interference that we describe here may constitute our strongest evolutionary conclusion. It potentially underscores the minor evolutionary advantage associated to CO interference at least in budding yeasts. In this context, we changed the title to be more factual and updated the text to better highlight the significance and implications of our findings.

**Major comments:**

1. The authors indicate that the distribution of hotspots and coldspots is not preserved between species, but this finding is not properly documented. I think it would be useful to include recombination maps in a main figure for all species (or at least for *S. cerevisiae*, *K. lactis* and *L. waltii*) with the elements highlighted. This will allow for a visual illustration of the variability in the recombination landscape between the studied species.

[R] The genomes of the species show blocks of synteny but overall, they are not collinear and therefore, it is not possible to have a direct comparison of the recombination maps. In our previous work, we have highlighted the non-conservation of CO hotspots between *S. cerevisiae*, *L. kluyveri* and *L. waltii* (Brion et al. 2017; Dutreux et al. 2023). Briefly, we retrieved conserved syntenic blocks in *L. kluyveri* and L. waltii genomes containing at least two S. cerevisiae orthologs associated with one hotspot.  L. waltii shares only five out of the 92 *S. cerevisiae* crossover hotspots (RHO5, SLS1, GYP6, OLE1 and MRPL8), while *L. kluyveri* shares only one. *L. waltii* and *L. kluyveri* share no crossover hotspots. In addition, our current study shows that none of the *K. lactis* hotspot is conserved in any of the four other species (response figure 1 and new supplementary figure S11).

**Response Figure 1**. Density of crossovers along the genome using a 5 kb window in the *S. cerevisiae* genome (Mancera et al. 2008; Oke et al. 2014; Krishnaprasad et al. 2015 combined dataset). Horizontal dotted green line represents crossover hotspot significance threshold. Solid spheres represent the conserved CO hotspots with either *L. kluyveri* (red) *or L. waltii* (blue). None of the 92 *S. cerevisiae* crossover hotspot is conserved in *L. lactis*.

2. Although analyses analogous to those presented in Fig. S5 had already been published in other comparisons of the recombination landscape in yeast (e.g. Dutreux et al., 2023), I think that Figs. S5A and S5B are worth to be presented in the main figures (not supplementary data). In many species of eukaryotes, the detection of NCOs is practically impossible, therefore only results for COs are presented. Therefore, it is perhaps also worth discussing the fact that the relationship applies to all recombination events and not only COs, and therefore is related to the regulation of DSBs frequency and not individual DSBs repair pathways.

[R] Figures S5A-B are now included in the main figure, Figure 2B. The association holds true for all total recombination (CO+NCO) events as well, new supplementary figure S6A.

3. The authors find that CO coldspots were associated with DNA repair genes. Unfortunately, an equivalent analysis was not performed for all recombination events (CO + NCO). I presume this approach is based on the belief that COs are more mutagenic than NCOs. However, recent studies in humans suggest that, at least in mammals, meiotic DSBs themselves are mutagenic, regardless of the pathway used for their repair (Hinch et al., Science 2023). Therefore, I would suggest repeating the analysis also considering NCOs (although I am aware that the picture of NCOs may be incomplete). I would also like to see some graphical representation of the analysis. Is it possible to perform a classic analysis of coldspots/hotspot enrichment in relation to gene ontology?

[R] As suggested, we performed the analysis to independently detect coldspots for all recombination events (CO+NCO). Based on a threshold of <2 events, we identified 20 coldspots for all recombination events (CO+NCO), i.e. 90% (18/20) of the CO+NCO coldspots were CO coldspots as well. The two additional coldspots that we identified were adjacent to the CO coldspots on end of the right arm of chromosome 5.

4. In relation to the previous point - it may be worth repeating this type of analysis also for other yeasts used in this study, or at least for S. cerevisiae, to be able to consider the extent to which this relationship is universal and dependent on the meiotic DSB repair pathway.

[R] The analysis regarding the CO coldspots has been performed in the other species as well. As mentioned in the main text, although some overlap between CO coldspots and DNA repair genes has been observed in the other species as well, we observed a significant enrichment in *K. lactis* only, maybe because the dataset is larger than in the other species.

5. In Fig. S7, the point where WGD occurred is marked in the wrong place, or at least that is what the sentence in the text says ("The Lachancea and Kluyveromyces species branched from the Saccharomyces lineage more than 100 million years ago, before to the ancestral whole-genome duplication (WGD) event specific of the S. cerevisiae lineage").

[R] We regret the oversight and have corrected the figure.

6. The result presented in Fig. S8 is interesting and should be shown in the main figures. Perhaps it would be worth adding an illustration illustrating simple versus complex COs.

[R] The old Figure S8 is now a part of main Figure 2C-D with the illustrations describing the CO types.

7. The last part of the results includes an analysis of the evolutionary rates of the ZMM genes. In the discussion, the authors should also refer the results of this analysis to the previous analysis of the overrepresentation of DNA repair genes in recombination coldspots. I understand that ZMM are not DNA repair proteins in the strict sense, but I think it is worth familiarizing readers with the authors' view on this matter. Moreover, I would suggest showing where MLH1 and MLH3 are located on the plot in Fig. 6 (especially the meiosis-specific MLH3), whether the selection pressure acts on them as on ZMM proteins, or rather as on DNA repair proteins. Showing the SLX4 and MUS81 would also be interesting.

[R] Figure 6 has been updated as suggested and now shows the *Mlh1*, *Mlh3, Slx4* and *Mus81* dN/dS values for the three species.

8. I feel like the discussion is underdeveloped. I missed a deeper summary of the comparison between meiotic recombination among the tested budding yeasts in the context of the presence and absence of functional ZMM. Even the title of the work is not properly developed in the manuscript text. The analysis shows that it is not the presence of a functional ZMM pathway or its lack that introduces differences between the individual recombination landscapes, although ZMM determines the presence of proper CO interference. With the caveat that for *L. kluyveri* it is basically unknown whether it has a functional ZMM or not. Maybe confirming the lack of expression of some ZMM genes in meiosis of this species would answer the question of how it should be treated?

[R] We agree with this reviewer that our original title was imprecise, so we changed it to be more factual, emphasizing on the multiple losses of crossover interference in budding yeasts. As stated above, it potentially underscores the minor/negligible evolutionary advantage associated to CO interference at least in budding yeasts. From there, it is hard to draw deeper conclusions since the actual roles/functions of CO interference are still under debate, notably in yeasts where the CO frequency tends to be high. We improved the discussion to better highlight these points.

We also agree that a deeper characterization of the ZMM factors persisting in the non-*Saccharomyces* yeasts would be informative, but we believe it is beyond the scope of the current manuscript and more suitable for a follow up work. However, our recent publication about *L. kluyveri* (Legrand et al 2024) shows that Zip3 is properly expressed in meiosis and behaves as in *S. cerevisiae* since it is located at DSB sites. Furthermore, we have unpublished transcriptomic data (Response Figure 2) showing that all the ZMM genes from *L. kluyveri* are specifically induced in meiosis (fold increase >16 at least compared to pre-sporulation conditions). Therefore, so far, although the level of CO interference in *L. kluyveri* is minimal, there is no indication that the ZMM genes are mis regulated.

**Response Figure 2.** Transcriptomic data showing that all the ZMM genes from *L. kluyveri* are specifically induced in meiosis (Unpublished data from Llorente Lab, CRCM, Marseille).

**Minor comments:**

1. In general, Figure captions are imprecise, many of them lack clear information explaining what is depicted. Authors should remember that figure legends should be self-sufficient.

[R] The figure legends have been updated and are now self-sufficient.

2. In the revised manuscript, I would suggest placing figure numbers on the figures and using line numbering, which would facilitate the reception of the work and possible reference to its individual elements in the review.

[R] We regret the omission. Figure numbers, Line numbers and Page numbers have been added.

Reviewer #1 (Significance (Required)):

The study provides a new insight into the variation in recombination landscape within budding yeast species with a special emphasis on crossover control. This includes also de novo assemblies of Kluyveromyces lactis genome and high-resolution tetrad-based maps of meiotic recombination events. Previously, recombination maps of different yeast species were compared, however this study focuses on budding yeasts, some of which lost ZMM pathway and differ in some crossover parameters, like interference and homeostasis. Although the analysis is interesting, it lacks the general conclusions regarding the evolution of recombination and the broader perspective that would be expected from a comparison of these phenomena in budding yeasts.


**Reviewer #2 (Evidence, reproducibility and clarity (Required)):**

This paper describes the genome-wide mapping of meiotic recombination in non-Saccharomyces yeast, Kluyveromyces lactis. By using heterologous parental strains, the authors mapped crossovers (COs) and noncrossovers (NCOs) on the genome of K. lactis which lacks proteins necessary for CO formation such as S. cerevisiae, mammals and plants. This is an extension of previous works by the authors’ group which mapped CO and NCO in different yeast, Lachancea kluyveri and L. waltii by a similar approach. The authors found that CO frequencies in K. lactis are much lower than those in S. cerevisiae and COs showed weaker interference, which facilitates the non-random distribution of COs along a chromosome. Overall, the experiments and informatic analyses have been done in good quality and the results are convincing. The paper provides additional new information on the landscape of meiotic recombination in different yeast species. These results are of great interest to researchers in the field of meiotic recombination and evolution of meiosis. There are some issues that the authors may be able to address before the publication.

Major points:
While the authors noted that K. lactic shows the loss of a pro-CO factors (ZMM protein), Spo16, and Msh5 (due to the introduction of an in-frame stop codon), it still possesses other proteins such as Zip1, Zip2, Zip3, Zip4/Spo22, Mer3, and Msh4. It is still likely that these pro-CO factors control CO formation (and interference) in this yeast. It would be nice for the authors to study whether the knockout of these genes is dispensable for CO formation and interference in meiosis. A similar analysis should be done for L. kluyveri which retains all ZMM genes, but this is clearly out of the scope of this paper.

[R] The question of the functions of the remaining ZMM factors is indeed interesting and related to point #8 from reviewer 1 (please see above). Although this is beyond the scope of our work, we would like to refer here to work from Amy McQueen’s lab using *L. lactis* Zip1 in *S. cerevisiae* (Voelkel-Meiman 2015). This study shows that *L. lactis* Zip1 does not allow synaptonemal complex assembly in *S. cerevisiae* but allows CO formation independently of the Msh4/5 complex but that depend on Zip2/4/Spo16 and Mlh1/3 for their resolution. Overall, these results suggests that *L. lactis* Zip1 at least retained ancestral functions shared with *S. cerevisiae* Zip1. However, it is not possible to conclude if the lack of full complementation of *L. lactis* Zip1 in *S. cerevisiae* comes from functional divergence or simply by the inability of *L. lactis* Zip1 to function properly in a heterologous context.

**Minor points:**

1. No page number, no main Figure number. It is hard to review this paper.

[R] We regret the oversight. Figure numbers, Line numbers and Page numbers have been added.

2. References: In some cases, in the Introduction, the authors referred to review papers such as Pyatnitskaya et al. (2019) for ZMM proteins while in the other parts, they referred to original papers; for example, three papers for Mlh1-Mlh3. If the number of references is not limited, original papers should be cited in the text.

[R] We regret this omission. Original papers have now been included in the citations.

3. Figure 3A, page 9, second paragraph: When the authors compared CO and NCO densities, it would be nice to show P-values for the comparison.

[R] p-values have now been added to the updated figure.

4. Please show a ratio of CO to NCO in each yeast in Figure 3B in the second paragraph of page 9 in the main text.

[R] The ratios have now been included in the figure for both the CO:NCO ratios and CO:corrected_NCO ratios, in the main text and figure legends.

5. Figure S5 and page 7, the first paragraph and page 9, third paragraph: CO/NCO densities (negative correlation to chromosome sizes) in S. cerevisiae should be checked with or without short chromosomes (I, III, and VI), which show very unique regulation of meiotic DSB formation (see Murakami et al. Nature 2020).

[R] Even excluding the small chromosomes, the size dependent trend persists for *S. cerevisiae* and *S. paradoxus*.

6. Table S7: Please add the S. cerevisiae gene name such as ZIP1 next to S. cerevisiae orthologs such as YDR285W. Moreover, please explain the column in detail or clarify the data. What does "meiosis" mean here? For example, YJL074C is SMC3, which is expressed in mitosis as well as in meiosis. The same is true for YGL163C, which is RAD54, which plays a minor role in meiosis, but plays a critical in mitotic DSB repair.

[R] We corrected Table S7 as desired by systematically including the standardized gene names.

The Gene Ontology (GO) annotation is a statement about the function of a particular gene. It offers a structured framework and a comprehensive set of concepts to describe the functions of gene products across all organisms. It is specifically crafted to support the computational representation of biological systems. In our specific case, we only looked at genes with the gene ontology annotation “meiosis”. Together, these statements comprise a “snapshot” of current biological knowledge and is by no means absolute. This has been detailed in the supplementary Table S7.

Reviewer #2 (Significance (Required)):

This study provides the landscape of meiotic recombination in non-Saccharomyces yeast, Kluyveromyces lactis. The genome-wide recombination map in K. lactis shows lower crossover frequencies with weaker crossover interference than those in S. cerevisiae. Overall, the experiments and informatic analyses have been done in good quality and the results are convincing. The paper provides additional new information on the landscape of meiotic recombination in different yeast species, particularly in terms of the evolution of meiotic recombination. These results are of great interest to researchers in the field of meiotic recombination and evolution of meiosis.


**Reviewer #3 (Evidence, reproducibility and clarity (Required)):**

Dutta et al. have compiled a genome-wide meiotic recombination map for Kluyveromyces lactis and compared it to a compilation of meiotic recombination maps for four other species, two of which (*Lachancea kluyveri* and *Lachancea waltii*), like *K. lactis*, predate the genome duplication event that produced the other two (Saccharomyces cerevisiae and S. paradoxus). Meiosis in many species studied (including metazoans and plants) shows control over the number and distribution of crossovers, which are critical for faithful chromosome segregation during meiosis. This takes the form of crossover interference, where crossovers are spaced more evenly than expected by chance, and crossover homeostasis, where many fewer chromosomes lack a crossover than is expected by chance. While both of the post-duplication species show both crossover interference and homeostasis, none of the pre-duplication species show crossover homeostasis, and crossover interference is very weak. In two cases (K. lactis and L. waltii), this can be explained by mutational loss of a few of the genes (called the ZMM genes) that promote meiotic crossovers in many species. However, *L. kluyveri* behavior cannot be explained in this way. Recombination hotspots are present but are not shared between the pre-duplication species or between the pre- and post-duplication species, perhaps not surprising for species that diverged more that 100 million years ago. Overall, this work will be a useful contribution to our understanding of the different possible flavors of meiotic recombination mechanisms and control that are possible (and, one might add, promote long-term species viability).
A) Evaluation, reproducibility and clarity
The work presented in this paper is straightforward and unimpeachable and will largely be of interest to those studying meiotic recombination, be it mechanistic studies or studies of the implications for population genetics. The analysis is technically correct, although there are some aspects where a slightly different emphasis should be considered (see comments below). However, the data and the analysis could stand as they currently are, without further revision.

Suggestions are below.
1. (trivial) it would have been useful if pages and lines were numbered.

[R] We regret the oversight. Figure numbers, Line numbers and Page numbers have been added.

2. "Across the 205 meioses...". In general, it would be desirable to apply compensation for the fact that NCOs and COs are differently detected. Since, in K. lactis, 35% of COs are not accompanied by detectable gene conversion, it seems reasonable to apply a correction to measured NCOs here and throughout the paper, regardless of the species. For example, if one assumes that 35% of NCOs are not detected, how does this affect estimates of chromosomes that do not appear to have undergone interhomolog recombination? Estimates of CO/NCO bias? In a similar vein, if the CO event is not considered (just the conversion events associated with it), how does this affect measures of conversion tract lengths in COs and NCOs?

[R] We thank the reviewer for this suggestion. We have performed the correction for the NCO estimates as described in Mancera et al. 2008, on a per tetrad basis across all the species. The fraction of missed NCOs were 7%, 34%, 30%, 23% and 25% respectively for *S. paradoxus*, *S. cerevisiae*, *K. lactis*, *L. waltii* and *L. kluyveri*. The fraction of missed NCOs depend upon the parental marker density~~.~~ In addition, we performed the CO:NCO bias analysis both with the detected and the corrected NCO frequencies and the trends remain unchanged (Now included in figure 3). Finally, we refrain from using the corrected NCO frequencies while reporting the NCO frequencies (Table 1, main text) to maintain uniformity with our previous work and since, these corrections do not alter any results.

3. It might be useful to report recombination event frequencies in terms of events/chromosome, as this, rather than event/unit distance, is functionally more relevant. In the same vein, it might be useful to consider total event homeostasis, in addition to just crossover homeostasis.

[R] This has been updated as suggested. <There is only CO homeostasis>.

4. An interesting observation is that two of the three pre-duplication species clearly at one time had a full complement of ZMM genes but lost some due to mutation. Have there ever been attempts to detect either synaptonemal complex or axial elements in these species?

[R] This is related to point #8 from reviewer 1 and to the major point of reviewer 2 (please see above).

To our knowledge, cytological observations of synaptonemal complex (SC) or axial elements have been performed in *L. kluyveri* only by us and the SC is clearly visible (Legrand et al 2024).

However, it is key to remind here that *K. lactis* axis protein encoding genes *HOP1* and *RED1* have been cloned by the Roeder’s lab by functional complementation of *S. cerevisiae* corresponding mutants, supporting the functional conservation of these genes (Smith and Roeder 2000). Finally, as mentioned above, *K. lactis* Zip1 retained at least some function of the ancestral Zip1 protein that are also shared by the *S. cerevisiae* protein (Voelkel-Meiman 2015).

5. The observation of elevated evolutionary rates in ZMM genes is also intriguing, but it would help if "dN/dS ratio" was defined.

[R] It is now defined in the text.

6. The observation of frequent E0 chromosomes is taken to suggest efficient achiasmate segregation; has the "corrected" NCO frequency been considered? Do the different frequencies of E0 chromosomes predict the different spore viabilities seen between species?

[R] E_0_ is not predictive at all of the spore viability as we have shown in previous studies (see *L. kluyveri* - Brion et al. 2017, *L. waltii* -Dutreux et al. 2023). In addition, this has been shown is *S. cerevisiae* as well (Nishant et al. 2009).

7. Figure 3A-what would this look like if it were plotted as "Events per chromosome" rather than per megabase?

[R] We changed the figure (now figure 2A) and plotted as events per chromosome to show the variability of events at the chromosome level.

8. Figure legends tend to be unreasonably terse, which makes figures more difficult to interpret.

[R] This has been updated as suggested.
