# Supplementary material for: Multiple independent losses of crossover interference during yeast evolutionary history
Source: PLoS Genet. 2024 Sep 26;20(9):e1011426. doi: 10.1371/journal.pgen.1011426 (PMC11460703; doi:10.1371/journal.pgen.1011426)
Supplement: Attachment — Submitted filename: Reviewers_response.docx [file pgen.1011426.s020.docx]

2^nd^ September, 2024

Dear Editor,

Please find enclosed the revised manuscript titled “Multiple independent losses of crossover interference during yeast evolutionary history” by Abhishek Dutta, Fabien Dutreux, Marion Garin, Claudia Caradec, Anne Friedrich, Gauthier Brach, Pia Thiele, Maxime Gaudin, Bertrand Llorente and Joseph Schacherer for consideration.

We appreciated the positive comments of the reviewers as well as the constructive feedback. We have incorporated changes to address the points raised by reviewers and revised the manuscript. The responses are attached below.

Regards,

Bertrand and Joseph

###############################################################

**Responses**

**Reviewer #1:** I reviewed this manuscript when submitted in Review Commons. The authors addressed all my minor comments properly.
As mentioned in my major comment, one issue I have in mind is the functions of ZMM genes in the meiosis of *Kluyveromyces lactis* (such as ZIP1, -2, -3, -4, MSH5 and MER3). Are these genes necessary for the formation of meiotic crossovers or not? If the authors’ idea is correct, it is unlikely that these ZMM genes promote the crossing-over. This could be simply tested by the construction of the deletion mutant of the genes (not complementation assay using *S. cerevisiae*) and checking the spore viability of the mutants as a minimum analysis of meiotic phenotypes. As in the response to the #1 reviewer, the authors showed some of these *K. lactis* genes are indeed induced during meiosis. This reinforces the idea of performing the analysis. But as I originally wrote, this is a bit out of the focus of the current version of this paper.

**Response:** We thank the reviewer for his comments. We agree the analysis of the ZMM mutants would be of great interest but this is indeed beyond the scope of this work and would be more suitable for a follow up.

**Reviewer #2:** The authors have done an excellent job of addressing my previous review comments, and I support publication once the numerical data underlying graphs are included.

**Response:** We thank the reviewer for their comments. All the additional raw data used to generate the plots have now been included in tables S3 and S5.
